# The role of mental disorders in precision medicine for diabetes: a narrative review

**DOI:** 10.1007/s00125-022-05738-x

**Published:** 2022-06-22

**Authors:** Sanne H. M. Kremers, Sarah H. Wild, Petra J. M. Elders, Joline W. J. Beulens, David J. T. Campbell, Frans Pouwer, Nanna Lindekilde, Maartje de Wit, Cathy Lloyd, Femke Rutters

**Affiliations:** 1 Epidemiology and Data Science, Amsterdam UMC location Vrije Universiteit Amsterdam, Amsterdam, the Netherlands; 2 Amsterdam Public Health Research Institute, Amsterdam, the Netherlands; 3 Usher Institute, College of Medicine and Veterinary Medicine, University of Edinburgh, Edinburgh, UK; 4 General Practice, Amsterdam UMC location Vrije Universiteit Amsterdam, Amsterdam, the Netherlands; 5 Julius Centre for Health Sciences and Primary Care, University Medical Centre Utrecht, Utrecht, the Netherlands; 6 Department of Medicine, University of Calgary Cumming School of Medicine, Calgary, AB Canada; 7 Department of Community Health Sciences, University of Calgary Cumming School of Medicine, Calgary, AB Canada; 8 Department of Cardiac Sciences, University of Calgary Cumming School of Medicine, Calgary, AB Canada; 9 Department of Psychology, University of Southern Denmark, Odense, Denmark; 10 Steno Diabetes Center Odense, Odense University Hospital, Odense, Denmark; 11 School of Psychology, Deakin University, Geelong, Australia; 12 Medical Psychology, Amsterdam UMC location Vrije Universiteit Amsterdam, Amsterdam, the Netherlands; 13 School of Health, Wellbeing and Social Care, Faculty of Wellbeing, Education and Language Studies, Open University, Milton Keynes, UK

**Keywords:** Mental disorders, Monitoring, Personalised medicine, Precision medicine, Prevention, Prognostics, Review, Treatment, Type 1 diabetes, Type 2 diabetes

## Abstract

This narrative review aims to examine the value of addressing mental disorders as part of the care of people with type 1 and type 2 diabetes in terms of four components of precision medicine. First, we review the empirical literature on the role of common mental disorders in the development and outcomes of diabetes (precision prevention and prognostics). We then review interventions that can address mental disorders in individuals with diabetes or at risk of diabetes (precision treatment) and highlight recent studies that have used novel methods to individualise interventions, in person and through applications, based on mental disorders. Additionally, we discuss the use of detailed assessment of mental disorders using, for example, mobile health technologies (precision monitoring). Finally, we discuss future directions in research and practice and challenges to addressing mental disorders as a factor in precision medicine for diabetes. This review shows that several mental disorders are associated with a higher risk of type 2 diabetes and its complications, while there is suggestive evidence indicating that treating some mental disorders could contribute to the prevention of diabetes and improve diabetes outcomes. Using technologically enabled solutions to identify mental disorders could help individuals who stand to benefit from particular treatments. However, there are considerable gaps in knowledge and several challenges to be met before we can stratify treatment recommendations based on mental disorders. Overall, this review demonstrates that addressing mental disorders as a facet of precision medicine could have considerable value for routine diabetes care and has the potential to improve diabetes outcomes.

**Graphical abstract:**

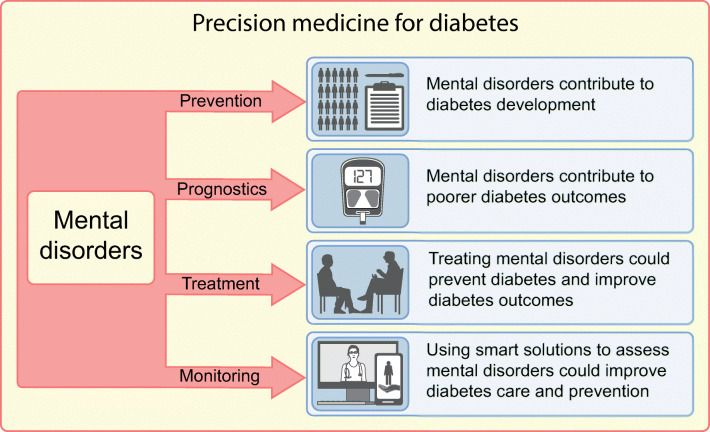

**Supplementary Information:**

The online version contains a slideset of the figures for download, which is available at 10.1007/s00125-022-05738-x.

## Introduction

The concept of precision medicine is often defined as providing the right therapy for individuals, in contrast to traditional approaches, which involve the development and implementation of interventions for groups and which do not consider individual variation in effectiveness [1]. However, up to now, the focus of precision medicine for diabetes care has been almost exclusively on biological factors, such as genetics or -omics. We hypothesise that there is a major potential for mental disorders to be considered as a facet of the prevention, prognostics, treatment and monitoring of diabetes and its outcomes. For the current review we used the Diagnostic and statistical manual of mental disorders (DSM)-5 definition of mental disorders [2] and included neurodevelopmental disorders (intellectual disability), schizophrenia and psychosis, bipolar disorder, depression, anxiety, obsessive–compulsive disorders, trauma- and stress-related disorders, feeding and eating disorders (anorexia, bulimia nervosa and binge-eating disorders), sleep–wake disorders (sleep disorders in general and insomnia), substance-related and addictive disorders and neurocognitive disorders (dementia). For the following DSM-5-defined mental disorders no evidence was found on their role as risk factors for diabetes and its complications and thus they are not discussed further: dissociative disorders, somatic symptoms and related disorders, gender dysphoria, elimination disorders, sexual dysfunction, disruptive impulse control and conduct disorders, personality disorders and paraphilic disorders. For some mental disorders opposite associations have been shown, for example diabetes leading to sexual dysfunction [3]. We omitted highly related mental health problems (i.e. burnout, work stress) and diabetes-specific mental health problems (e.g. diabetes burnout, diabetes distress, fear of hypoglycaemia) and focused only on DSM-5-defined mental disorders. However, diabetes-specific mental health issues could also be an interesting target for precision medicine in diabetes. For example, diabetes burnout and diabetes distress lead to suboptimal diabetes self-management and poor diabetes outcomes, and diabetes distress-tailored psychological interventions have been shown to effectively improve blood glucose levels [4, 5].

In our umbrella review of 32 systematic reviews, we showed that comorbidity of diabetes and mental disorders is common. The prevalence of type 2 diabetes varied between 8% and 40% for different mental disorders [6]. The prevalence of mental disorders is also high among people with diabetes. For example, women with type 1 diabetes have a 2-fold increased risk for developing an eating disorder [7] and a 2.3-fold higher prevalence of gender dysphoria [8]. Autism, personality disorders, intermittent explosive disorder and obsessive–compulsive disorder are also more prevalent in people with diabetes [9–13]. Overall, these results show a bidirectional association between mental disorders and diabetes [14]. However, for simplicity and because of limited space, in this narrative review we focus on mental disorders as a facet of diabetes precision medicine. Our review aims to examine the potential value of addressing mental disorders as a facet of all four key aspects of precision medicine for diabetes: prevention, prognostics, treatment and monitoring [1]. We use the term ‘diabetes’ to refer to both type 1 diabetes and type 2 diabetes and to diabetes of undefined aetiology. If data are specific to type 1 or type 2 diabetes, this is specified.

## Mental disorders and diabetes incidence and outcomes (precision prevention and prognostics)

### Mental disorders and diabetes incidence

In the last few years, substantial evidence has been gathered on the association between mental disorders and the risk of developing type 2 diabetes. For example, in Scotland, type 2 diabetes incidence was 1.5- to 2.5-fold higher in people with a history of hospitalisation for schizophrenia, depression or bipolar disorder than in those without a history of hospitalisation for these disorders [15]. In the Nurses’ Health Study II, post-traumatic stress disorder was associated with a 1.8-fold higher risk (HR 1.80 [95% CI 1.50, 2.10]) of incident type 2 diabetes [16]. Our recent umbrella review summarised the findings from 25 systematic reviews, showing robust associations between four mental disorders and diabetes in adults [14]. For incident type 2 diabetes we reported an RR ranging from 1.18 (95% CI 1.12, 1.24) to 1.60 (95% CI 1.37, 1.88) for depression, an RR ranging from 1.55 (95% CI 1.21, 1.99) to 1.74 (95% CI 1.30, 2.34) for insomnia, and an OR of 1.47 (95% CI 1.23, 1.75) for anxiety disorders [14]; individual studies reported an RR of 1.70 (95% CI 1.20, 2.50) for bulimia nervosa, an OR of 3.34 (95% CI 0.85, 13.12) for binge-eating disorder [17] and an RR of 2.53 (95% CI 1.70, 3.80) for schizophrenia [18]. Compared with the general population, people with an intellectual disability or substance abuse disorder may be at greater risk of diabetes (effect sizes not reported) [19, 20]. Finally, we did not identify any studies on the association between mental disorders and incidence of type 1 diabetes.

There is little evidence that neurocognitive disorders are causally related to the incidence of diabetes. However, the reverse is probably true: a recent meta-analysis concluded that diabetes conferred a 1.25- to 1.91-fold excess risk of cognitive disorders (both cognitive impairment and dementia), and elevated levels of 2 h glucose and HbA_1c_ and low and high levels of fasting plasma insulin were associated with an increased risk of dementia in people with (or with an increased risk of) diabetes [21]. Dementia was defined as all-cause dementia, Alzheimer’s disease or vascular dementia, corresponding to the DSM-5 definition of major neurocognitive disorder. Cognitive impairment was defined as mild cognitive impairment, corresponding to the DSM-5 definition of mild neurocognitive disorder, or deficits of memory, executive function, processing function, language, attention, visuospatial ability and reasoning [2].

**Fig. 1:**
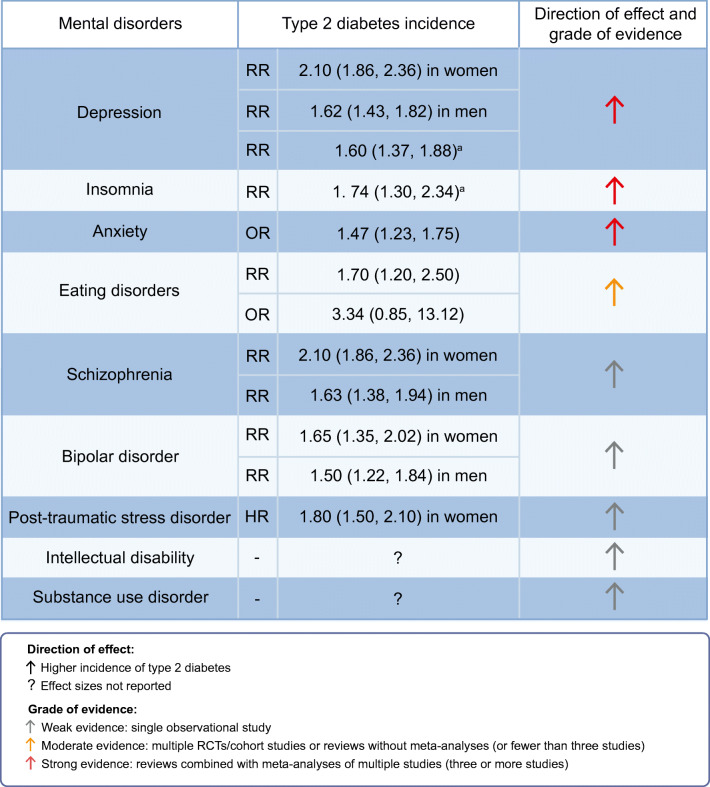
Summary of the association between mental disorders and incidence of type 2 diabetes (precision prevention). Effect sizes are given as OR (95% CI), RR (95% CI) or HR (95% CI). To our knowledge, no studies have described the association between mental disorders and incidence of type 1 diabetes. We found no studies describing the association of obsessive–compulsive disorders, neurocognitive disorders (dementia), dissociative disorders, somatic symptoms and related disorders, gender dysphoria, elimination disorders, sexual dysfunction, disruptive impulse control and conduct disorders, personality disorders and paraphilic disorders with incidence of diabetes. ^a^This umbrella review reported ranges of effect sizes [14]; the upper range effect size is reported here for simplicity. This figure is available as part of a downloadable slideset

Figure 1 provides a summary of the associations between mental disorders and incidence of type 2 diabetes, including an assessment of the grade of evidence from the studies included in this review. In summary, there is quite strong evidence from large observational population-based prospective studies, with some evidence summarised in meta-analyses, that some mental disorders are associated with a higher incidence of type 2 diabetes and thus could be a target for precision prevention.

### Mental disorders and diabetes outcomes

Mental disorders also play a role in diabetes outcomes, resulting in poorer blood glucose levels and microvascular and macrovascular complications. A meta-analysis showed that, in people with diabetes, substance abuse was associated with higher blood glucose levels [22]. Similarly, a meta-analysis of longitudinal studies from our group showed that, in people with diabetes, depression was associated with an increased risk of incident macrovascular complications (HR 1.38 [95% CI 1.30, 1.47]) and microvascular complications (HR 1.33 [95% CI 1.25, 1.41]) [23]. We identified one cross-sectional study on anxiety and diabetes, performed in an Australian eye hospital, which showed no association of anxiety with diabetes-related complications (e.g. retinopathy) [24]. A prospective study (1 year of follow-up) of 131 people with type 2 diabetes and obsessive–compulsive behaviour showed higher levels of HbA_1c_ (>53 mmol/mol) in those who scored higher for obsessive–compulsive behaviour [25]. Additionally, a cross-sectional study in male veterans with diabetes found higher cholesterol and LDL-cholesterol levels in those with post-traumatic stress disorder and depression [26].

Prospective German, Austrian and Canadian studies among people with type 1 diabetes (aged 8–30 years) observed associations between eating disorders and higher rates of ketoacidosis and hyperglycaemia [27] and impaired metabolic control and diabetic retinopathy [28]. These associations are possibly related to insulin restriction in people with type 1 diabetes who want to lose weight, affecting both blood glucose levels and risk of complications [29]. A cross-sectional study in 152 patients from a Turkish diabetes outpatient clinic showed no association between binge-eating disorder and blood glucose levels in people with type 2 diabetes [30]. Additionally, a large cross-sectional data linkage study showed that people with diabetes and intellectual disorders were less likely to have cardiovascular complications than people with diabetes and no intellectual disorder in the Dutch population, while diabetes-related surgical interventions (undefined) and hospitalisation occurred more often in those with diabetes and intellectual disorders [31]. Furthermore, in our meta-analysis of 78 mainly cross-sectional studies, we showed that, in people with type 2 diabetes, those with insomnia had higher HbA_1c_ levels than those without insomnia (mean difference [MD] 0.23% [95% CI 0.10, 0.40]) [32]. Finally, a population-based case–control study performed in Minnesota showed that a diagnosis of dementia in people with diabetes resulted in a higher OR of 1.80 (95% CI 1.13, 2.89) for the presence of diabetes complications, including neuropathy, retinopathy or nephropathy [33].

Overall, there is moderate evidence, consisting of cross-sectional studies and several prospective studies, with some evidence summarised in meta-analyses, suggesting that the mental disorders discussed in this section are associated with poorer diabetes outcomes and thus could be the target for precision prognostics.

## Prevention of diabetes and improvement of diabetes outcomes by treatment of mental disorders (precision treatment)

### Prevention of diabetes by treatment of mental disorders

To our knowledge, only one study has reported on the specific association between mental disorder treatment and incident diabetes. This study showed that, of 1598 people with post-traumatic stress disorder, those with clinically meaningful improvements in symptoms had a 49% lower risk of incident type 2 diabetes [34]. The lack of other studies represents a major gap in the literature. However, there are several examples of effective improvements in proxy measures of potential diabetes (e.g. fasting glucose and HbA_1c_) after treating mental disorders. The behavioural and pharmacological interventions described in this and the following section may affect glycaemic measures not only directly, but also indirectly through intersectional factors, such as health behaviours.

A meta-analysis of interventions for improving blood glucose levels (rather than treatment of mental disorders) in >40,000 people with schizophrenia, bipolar disorder or severe depression showed that pharmacological interventions (MD −0.11 mmol/l [95% CI −0.19, −0.02]) and behavioural interventions (MD −0.28 mmol/l [95% CI −0.43, −0.120]) were effective at lowering fasting glucose compared with control; in a subgroup analysis of pharmacological interventions, the interventions were also effective at lowering HbA_1c_ [35]. This meta-analysis included 28 studies investigating pharmacological interventions that might directly affect blood glucose levels, including diabetes medication (e.g. metformin), weight loss medication or other medication influencing metabolic function, and 11 studies that included behavioural interventions targeting lifestyle, weight loss or physical exercise. The meta-analysis included 21 studies that investigated interventions that might affect blood glucose levels indirectly, including switching to antipsychotic drugs with fewer metabolic side effects or treatment of mental disorders (e.g. antidepressants). Similarly, our group showed an indirect effect on fasting glucose (β=−0.65 mmol/l [95% CI −1.40, 0.02]) of a multidisciplinary lifestyle-enhancing treatment (based on improving daily structure and consisting of sports-related activities, work-related activities and psychoeducation) in 65 people with severe mental illness receiving long-term care in a psychiatric hospital (unknown diabetes status) [36].

A recent meta-analysis of treatments for sleep disorders found that, in two general population studies and one type 2 diabetes population study, behavioural inventions, including cognitive behavioural therapy (CBT) and sleep education, showed improved self-reported sleep quality but no indirect effect on blood glucose levels, as fasting glucose was not lowered (MD 0.12 mmol/l [95% CI −0.37, 0.12]) [37]. In addition, the majority of the pharmacological interventions included (e.g. suvorexant, eszopiclone and melatonin) showed improved sleep quality, but effects on diabetes-related outcomes were mixed, with either improving fasting glucose after treatment with sleep medication or no effect [37]. These studies indicate that pharmacological treatment of sleep disorders may improve glycaemic variables through indirect effects [37–39].

More studies are needed to assess the effectiveness of treatment for other DSM-5-defined mental disorders.

### Improvement of diabetes outcomes by treatment of mental disorders

The evidence on improving diabetes-related outcomes through treatment of mental disorders is inconsistent. Two meta-analyses on the effect of psychological interventions for mental disorders (e.g. CBT and counselling) on improvement of blood glucose levels and diabetes self-management showed a non-significant standardised mean difference (SMD) for HbA_1c_ of –0.12 (95% CI −0.27, 0.03) for adults with type 1 diabetes and a significant SMD of −0.19 (95% CI −0.25, −0.12) for adults with type 2 diabetes compared with control; for type 2 diabetes, this was equivalent to a reduction in HbA_1c_ of 3.7 mmol/mol, indicating that these psychological interventions also indirectly lower HbA_1c_ [40, 41]. No effect on diabetes self-management (e.g. self-monitoring blood glucose, medication intake, diet, physical activity) was demonstrated, partly because of a lack of studies suitable for meta-analysis. This suggests that psychological interventions have clinical benefits; however, this meta-analysis did not stratify results by type of treatment or by treatment indication [40, 41], making it hard to suggest a precision medicine approach, that is, what would work for whom. A recent meta-analysis of 43 RCTs on the treatment of depression in people with diabetes showed that all interventions had a significant effect on depressive symptoms compared with control, with significant beneficial effects on HbA_1c_ levels for pharmacological treatment, including indirectly through the use of antidepressants (SMD 0.99 [95% CI 0.13, 1.85]), group therapy (SMD 0.95 [95% CI 0.19, 1.72]), psychotherapy (SMD 0.61 [95% CI 0.15, 1.07]) and collaborative care (SMD 0.21 [95% CI 0.05, 0.36]) [42]. Baseline depression scores and HbA_1c_ modified the treatment effect, with higher baseline depression scores (measured using diverse questionnaires) associated with a greater reduction in HbA_1c_, suggesting a need for the precision application of these treatments [42]. Another review by our group showed that antidepressant use may, through an indirect effect, improve blood glucose levels in people with depression and reduce the risk of diabetic retinopathy in women, while a separate meta-analysis showed that there may be an increased risk of hospitalisation for hyperglycaemia in people using antipsychotics [43, 44].

In an analysis of four studies, three including people with type 2 diabetes and one in the general population, as part of the meta-analysis on sleep interventions mentioned in the previous section, the behavioural treatment of sleep disorders resulted in significantly improved self-reported sleep quality but did not significantly affect HbA_1c_ (MD −0.35% [95% CI −0.84, 0.13]) compared with control [37]. An RCT in people with psychosis, of whom 40% had schizophrenia, and suboptimal management of type 2 diabetes showed that a mental health centre collaborative care treatment approach (e.g. self-management of psychotic symptoms) to directly improve blood glucose levels, diabetes outcomes and self-management (including medication intake, nutrition and physical activity) resulted in an improvement in HbA_1c_ of –1.1% (*p*=0.049) compared with usual care [45]. Several initiatives have been proposed to decrease anxiety in those with diabetes, but no results have been presented yet [46]. With regard to the treatment of eating disorders with behavioural interventions (e.g. psychoeducation or CBT), a meta-analysis of three studies performed in Canada and Japan showed a non-significant SMD of –0.21 (95% CI −0.58, 0.16) for HbA_1c_ in women with type 1 diabetes compared with control [47].

Special mention should be made of e-health interventions, which may be a useful approach in the current context of the COVID-19 pandemic. An RCT of 255 people with depression and diabetes, performed in the Netherlands, showed that a higher percentage of the group undergoing web-based CBT than the control group experienced a reduction in depressive symptoms (41% vs 24%; *p*<0.001), but the intervention had no indirect beneficial effect on blood glucose levels [48]. Recent technological developments suggest that self-help applications might be an interesting approach for improving mental health in people with diabetes; however, to date no such studies have been conducted with individuals with diabetes and diagnosed mental disorders.

**Fig. 2:**
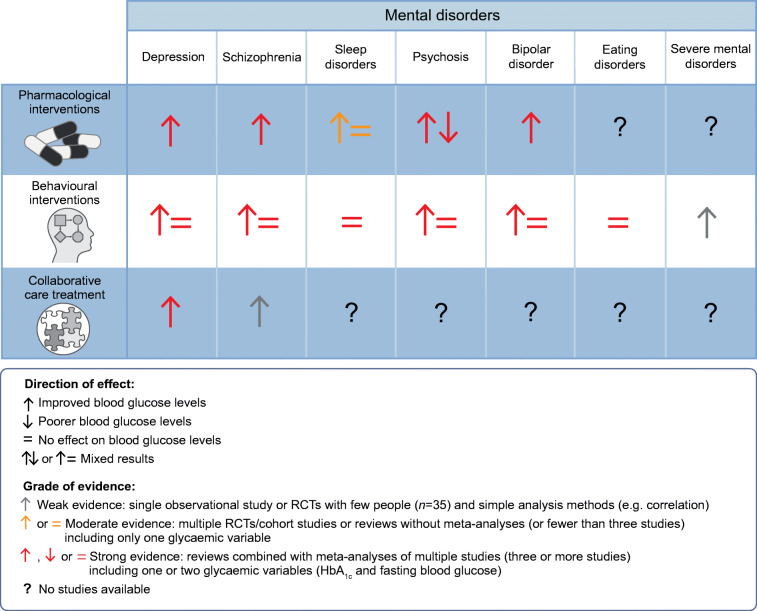
Summary of the effect of treatment for mental disorders on glycaemic variables in people at risk for or who have diabetes (precision treatment). To our knowledge, no studies have investigated the effect of treatment of mental disorders on glycaemic variables in people with an anxiety disorder, neurodevelopmental disorders (intellectual disability), substance use disorder, obsessive–compulsive disorders, trauma- and stress-related disorders, neurocognitive disorders (dementia), dissociative disorders, somatic symptoms and related disorders, gender dysphoria, elimination disorders, sexual dysfunction, disruptive impulse control and conduct disorders, personality disorders and paraphilic disorders. This figure is available as part of a downloadable slideset

Figure 2 provides a summary of the effects of treatment for mental disorders on glycaemic variables in people at risk for or who have diabetes; an assessment of the grade of evidence in the studies included in this review is also provided. For pharmacological and behavioural interventions, quite strong evidence from multiple meta-analyses and single studies showed an association with improved blood glucose levels in people with depression, schizophrenia, psychosis and bipolar disorder; however, for behavioural interventions an association was not always found for all glycaemic markers (e.g. improved fasting glucose but not HbA_1c_).

In summary, about 35% of the studies included in this section investigated pharmacological interventions that may directly affect blood glucose levels (e.g. diabetes medication), whereas the other studies investigated pharmacological treatments for mental disorders that may affect blood glucose levels indirectly (e.g. antidepressants). With regard to behavioural interventions, 18% of the included studies investigated interventions that may directly affect blood glucose levels (e.g. weight loss interventions), whereas 82% investigated interventions that may indirectly affect blood glucose levels (e.g. CBT for depression). The meta-analyses of depression and sleep disorders showed an association between improvement of symptoms and improved diabetes-related outcomes, aiding precision treatment [37, 42]. However, more research is needed as the evidence is inconsistent and no studies were found on the effects of treatment for other mental disorders. To our knowledge, no studies have considered heterogeneity in diabetes-related outcomes regarding treatment of mental disorders within the diabetes population. However, post hoc analyses show that this heterogeneity is present. For example, in a Dutch study from our group, we showed that treating depression with light therapy in people with type 2 diabetes was more effective in those with higher HbA_1c_ levels than in others [49]. We suspect that part of the reason for the limited use of the precision treatment approaches based on aspects of mental disorders is a lack of data on which to base this stratification.

## Detailed assessment of mental disorders (precision monitoring)

In contrast to the assessment of biological variables as part of routine diabetes care, the assessment of mental disorders was introduced only recently in speciality diabetes care [50, 51] and is seldom addressed, even within tertiary diabetes centres [52], despite recommendations in several guidelines [53, 54]. This limited implementation is related to the assessment being time-consuming and thus costly, as well as a lack of treatment options (e.g. long waiting lists for treatment). Moreover, current measures for the assessment of mental disorders lack precision and are thus difficult to use for precision monitoring and assessing within-person changes [50, 51]. However, recent technological developments may aid the more precise assessment of mental disorders in routine diabetes care. Computer adaptive testing (CAT) allows for the selection of specific questions based on the answer(s) to previous questions. For example, if individuals respond to a question on whether they have depressive feelings that they have no such feelings, a subsequent question on how often they have those feelings is omitted. By asking questions tailored to the responses of each individual, CAT reduces the patient and administrative burden by up to 75% compared with fixed-item questionnaires [55]. CAT questionnaires provide an opportunity to assess more domains with fewer questions and thus enable the more precise and straightforward measurement of mental disorders and monitoring of within-person changes over time [55]. CAT questionnaires on anxiety, depression, fatigue, sleep disturbance and pain are available and are being validated for people with diabetes in a current research project [56].

In addition to CAT questionnaires, ecological momentary assessment (EMA) may aid the assessment of mental disorders. EMA involves repeated sampling of participants’ behaviours and experiences in real time and in their natural environments, often using smartphone applications [57, 58]. For example, one may ask regular questions on depressive symptoms by performing multiple assessments over a relatively short period and thus provide a more representative picture of the course of a disorder. EMA allows for better evaluation of within-person changes, which in chronic diseases with high recurrence may provide valuable information for the patient, healthcare providers and researchers [59]. Several studies summarised in a systematic review carried out by our group used EMA methodology to link depression to glycaemic variables, showing that high variability in depressive symptoms was associated with higher glucose variability, especially in those with type 1 diabetes [60]. A recent review of studies using EMA in people with diabetes to assess stress, anxiety and depression showed that changes in these conditions predicted suboptimal diabetes self-management [59], suggesting that, despite limited research (only ten studies were included), EMA may have potential clinical utility for diabetes care. However, it is important to ensure that the use of such technologies does not widen health inequalities, as these technologies may not be widely available to all who need them. 
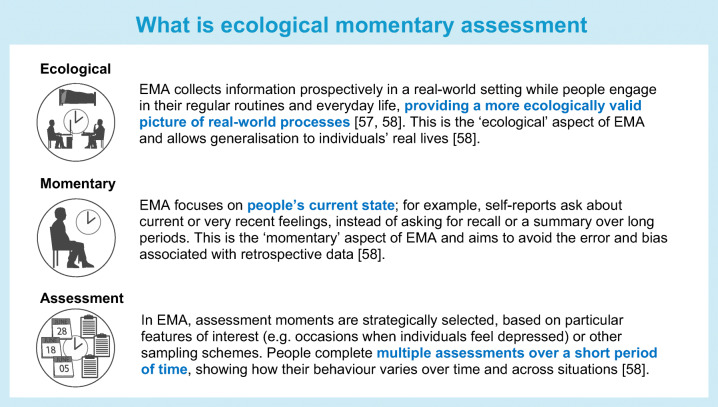


The integration of CAT and/or EMA in clinical practice may have the potential to improve care for people with diabetes, enabling screening, identifying and monitoring of mental disorders, guiding diabetes treatment and improving patient–clinician communication, and enabling people to become more involved in diabetes self-management. However, using only CAT and/or EMA may not affect diabetes outcomes: it is important not only to measure mental disorders, but also to discuss and act on the results of these measurements and treatments. For example, when depressive symptoms are identified, the next step is to conduct a diagnostic interview to establish whether a depressive disorder is present and its severity. Evidence-based national and international guidance will then dictate the individualised treatment pathway, which may include pharmacotherapy or evidence-based psychological treatment [42, 61, 62]. However, it needs to be recognised that measurement of mental disorders should not be performed in the absence of sufficient capacity and resources to provide cost-effective interventions, and that more research is needed to develop effective guidelines. For example, previous research has shown that the use of patient-reported outcome measures (PROMs) of general well-being followed by a discussion of outcomes improves psychological well-being in both adults and youth with diabetes, as well as improving clinical and cost outcomes [63]. Despite limited research regarding the clinical utility of CAT, EMA and PROMs for monitoring mental disorders in diabetes care because of the novelty of the topic (which has been summarised in only two narrative reviews), we believe that these technologically enabled solutions have the potential to aid the precision monitoring of diabetes.

## Discussion

In this narrative review we have discussed the potential value of addressing mental disorders as a facet of all four key aspects of precision medicine for diabetes, namely prevention, prognostics, treatment and monitoring. We have summarised the literature to date and provided food for thought on the concept of mental disorders and diabetes-specific precision medicine.

There were some methodological issues in the included studies. First, some of the findings are indirect or of limited scientific value as they are based on small observational studies, including only cross-sectional data or case–control data, making it difficult to determine the direction of the observed associations. For example, diabetes may be a risk factor for mental disorders such as dementia, while the treatment of diabetes with metformin is associated with a lower risk of dementia by causing slower cognitive decline [64]. Second, we found only one study that reported on the direct association between treatment of mental disorders and diabetes incidence; other studies reported only on the improvement of glycaemic variables. It is important to acknowledge that, as also described in the introduction, the evidence base for the role of several DSM-5-defined mental disorders as risk factors for the development of diabetes and its complications is limited and more research in the general population and with people with diabetes is needed. Third, we omitted highly related mental health problems, such as burnout and work stress, as space in this review was limited; we focused only on DSM-5-defined mental disorders [2]. However, one of our own meta-analyses shows a strong association between burnout and diabetes [65]. Similarly, we omitted diabetes-specific emotional problems, such as diabetes burnout, fear of hypoglycaemia, negative perceptions of insulin therapy, satisfaction with treatment and diabetes distress, which should also be considered in precision medicine in diabetes [66]. Fourth, the interventions described in the treatment section, such as group therapy, may affect people’s mental state not only directly but also indirectly by affecting health behaviours.

Moreover, we did not use medication prescription for mental disorders as a proxy measurement in this review because of methodological limitations; these medications are also prescribed for other conditions (e.g. pain, migraine and vomiting) and it is challenging to separate the contributions of the mental disorders themselves from the effects of treatments in observational studies. For example, there is evidence that some pharmacological treatments for mental disorders are associated with a higher risk of type 2 diabetes [43, 67–70]. The strongest evidence is for antipsychotics, which may increase the risk of type 2 diabetes indirectly (e.g. through weight gain) or directly by impairing insulin sensitivity [68]. In addition, antidepressant use has been observed to be a risk factor for type 2 diabetes development, independently of depression, with slightly higher risks in those using non-selective serotonin reuptake inhibitors (non-SSRIs) than in those using SSRIs [69, 71]. Sleep medications, such as melatonin and gamma-aminobutyric acid (GABA) receptor modulators, are also thought to have a negative effect on glycaemic control [70]. However, we may have missed evidence by not including medication prescription as a proxy, as several studies used this approach. For example, a meta-analysis by our group on risk of incident type 2 diabetes reported an OR ranging from 1.93 (1.37, 2.73) to 1.94 (95% CI 1.34, 2.80) for use of antipsychotic medication and a RR of 1.27 (95% CI 1.19, 1.35) and an OR of 1.50 (95 CI% 1.08, 2.10) for use of antidepressant medication [14]. In this review we present no data on the obvious role of other intersectional factors, such as social determinants of health (e.g. home environments, work place hazards, food security, financial stability) and health disparities, which all interact and play a role in diabetes precision medicine, as this is discussed in the review by Tuchman [72] in this special issue on precision medicine. Culture-related factors and geographical conditions should also be considered as facets of diabetes precision medicine [73]. For example, one review showed that, in indigenous people in Australia, New Zealand and South America, rapid changes in diet due to recent changes in the environment (e.g. reduced hunting grounds and food sources) are associated with a higher risk for diabetes [73]. Finally, diversity and inclusion in research participants should be a focus point, as people of non-European ancestry continue to be under-represented in precision medicine research [74].

There are multiple mechanisms that may explain the association between mental disorders and diabetes (outcomes). First, a cross-sectional study in three primary care clinics in Saudi Arabia showed that depression was significantly associated with lower trust in general practitioners [75], and a meta-analysis of 47 independent population samples showed that depression was significantly associated with suboptimal diabetes self-care (*z*=9.97; *p*=0.0001), including a less healthy diet, lower levels of exercise, less frequent glucose monitoring and missing medical appointments [76]. These mechanisms can be a successful point of intervention, as shown, for example, by a physical activity intervention trial in 100 adults living in a group home in South Africa with intellectual disabilities, who showed improvements in glucose after 12 weeks [77]. Second, veterans in the USA with diabetes and mental disorders, particularly substance abuse disorders, were less likely to receive recommended interventions such as retinopathy screening [78]. Third, medication provided to treat mental disorders can alter insulin sensitivity [43, 67–70]. Finally, biological mediators may play a role, such as cytokines, catecholamine, changes in neurogenesis/neurotransmitter metabolism, and hyperactivity of the hypothalamus–pituitary–adrenal axis [79].

### Future directions and challenges

This review has demonstrated the potential value of addressing mental disorders as a facet of diabetes precision medicine. The evidence that is summarised here and in the ADA consensus report [53] indicates that mental disorders should be considered a central aspect of precision medicine for diabetes, namely as part of prevention, prognostics, treatment and monitoring. Although assessment of mental disorders is embedded in several diabetes care guidelines, we hope that this narrative review will stimulate new innovative scientific contributions to the field and highlight the need for more precise methods of assessment (e.g. precision monitoring), thereby improving the implementation of the assessment of mental disorders in diabetes care.

However, it is also clear that there are considerable gaps in knowledge and several challenges to be met. First, for many DSM-5-defined mental disorders, it is still unknown whether treatment in those with diabetes improves blood glucose levels or reduces the risk of diabetes incidence and complications. Second, this narrative review discusses the relative grade of evidence and consistency of studies included (see Figs 1, 2). However, we believe that a systematic review of all currently available evidence is needed to further summarise and assess the quality of the evidence available. Third, we need to determine if mental disorders are more common in certain diabetes subgroups, as recently identified by Ahlqvist et al, including severe autoimmune diabetes, severe insulin-deficient diabetes, severe insulin-resistant diabetes, mild obesity-related diabetes and mild age-related diabetes [80]. Fourth, better quality evidence is needed to identify the cost-effectiveness of the treatment of mental disorders for the prevention of diabetes. Moreover, currently available RCTs mostly use a ‘one size fits all’ approach, providing people with diabetes with one treatment for mental disorders, instead of further individualising and identifying individual treatment needs. Finally, the biggest challenge is to establish sustainable human and financial resources to implement recommended treatments for mental disorders within usual care settings for people with diabetes.

### Conclusion

This narrative review has shown that several mental disorders are associated with a higher risk of type 2 diabetes and its complications, while there is suggestive evidence indicating that treating some mental disorders could contribute to the prevention of diabetes and improve diabetes outcomes. Using technologically enabled solutions to identify mental disorders could help individuals who stand to benefit from particular treatments. However, there are considerable gaps in knowledge and several challenges to be met before we can stratify treatment recommendations based on mental disorders. Overall, this narrative review indicates that addressing mental disorders as a facet of precision medicine could have considerable value for routine diabetes care and has the potential to improve diabetes outcomes.

## Supplementary information

ESM 1 (PPTX 224 kb)

## Abbreviations

CAT: Computer adaptive testing
CBT: Cognitive behavioural therapy
DSM: Diagnostic and statistical manual of mental disorders
EMA: Ecological momentary assessment
MD: Mean difference
PROM: Patient-reported outcome measure
SMD: Standardised mean difference
SSRI: Selective serotonin reuptake inhibitor
