# Supplementary material for: The role of mental disorders in precision medicine for diabetes: a narrative review
Source: Diabetologia. 2022 Jun 22;65(11):1895–906. doi: 10.1007/s00125-022-05738-x (PMC9213103; doi:10.1007/s00125-022-05738-x)
Supplement: Supplementary file 1 — (PPTX 224 kb) [file 125_2022_5738_MOESM1_ESM.pptx]

## Slide 1
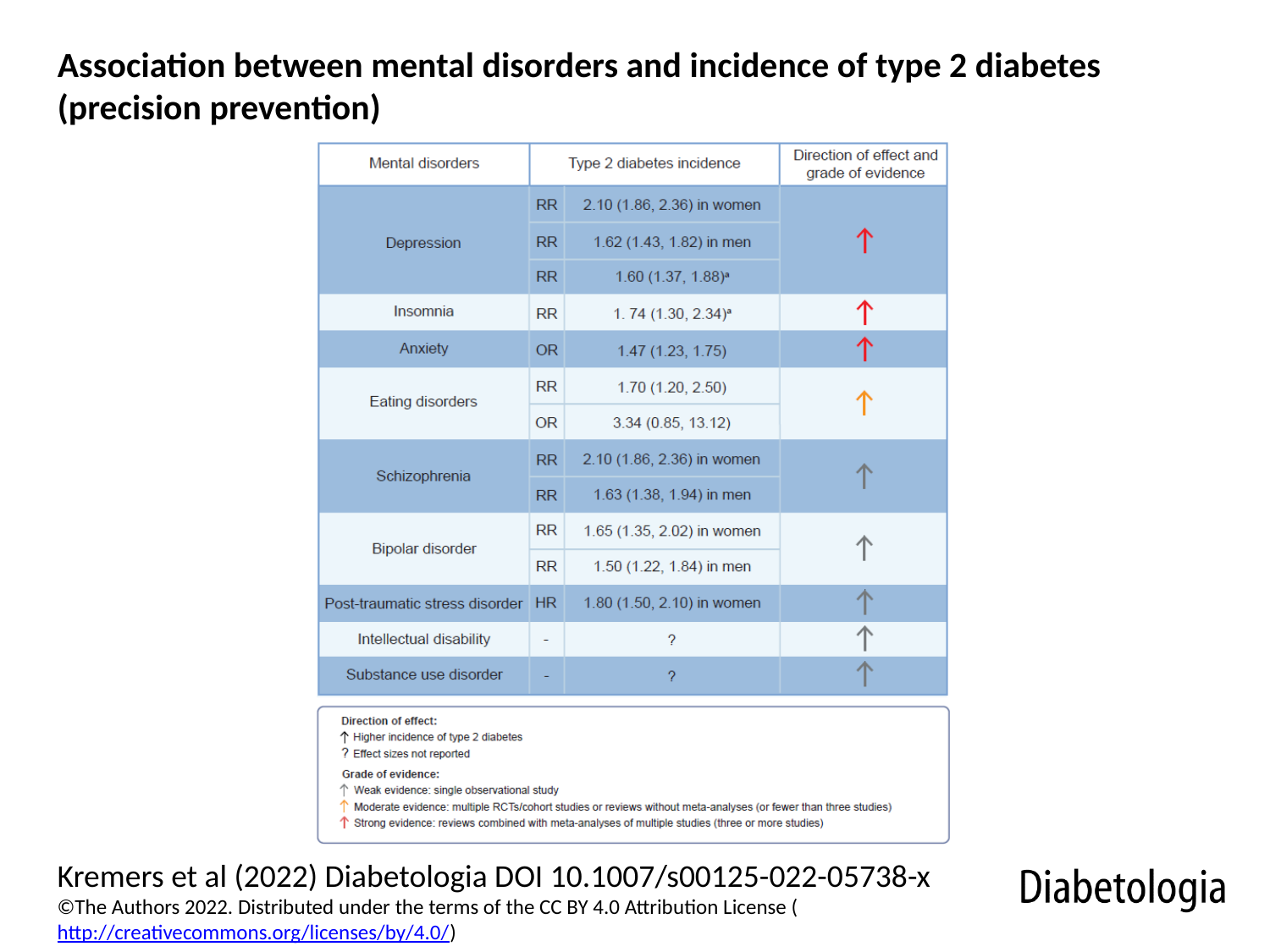

Association between mental disorders and incidence of type 2 diabetes (precision prevention)
Kremers et al (2022) Diabetologia DOI 10.1007/s00125-022-05738-x
©The Authors 2022. Distributed under the terms of the CC BY 4.0 Attribution License (http://creativecommons.org/licenses/by/4.0/)

## Slide 2
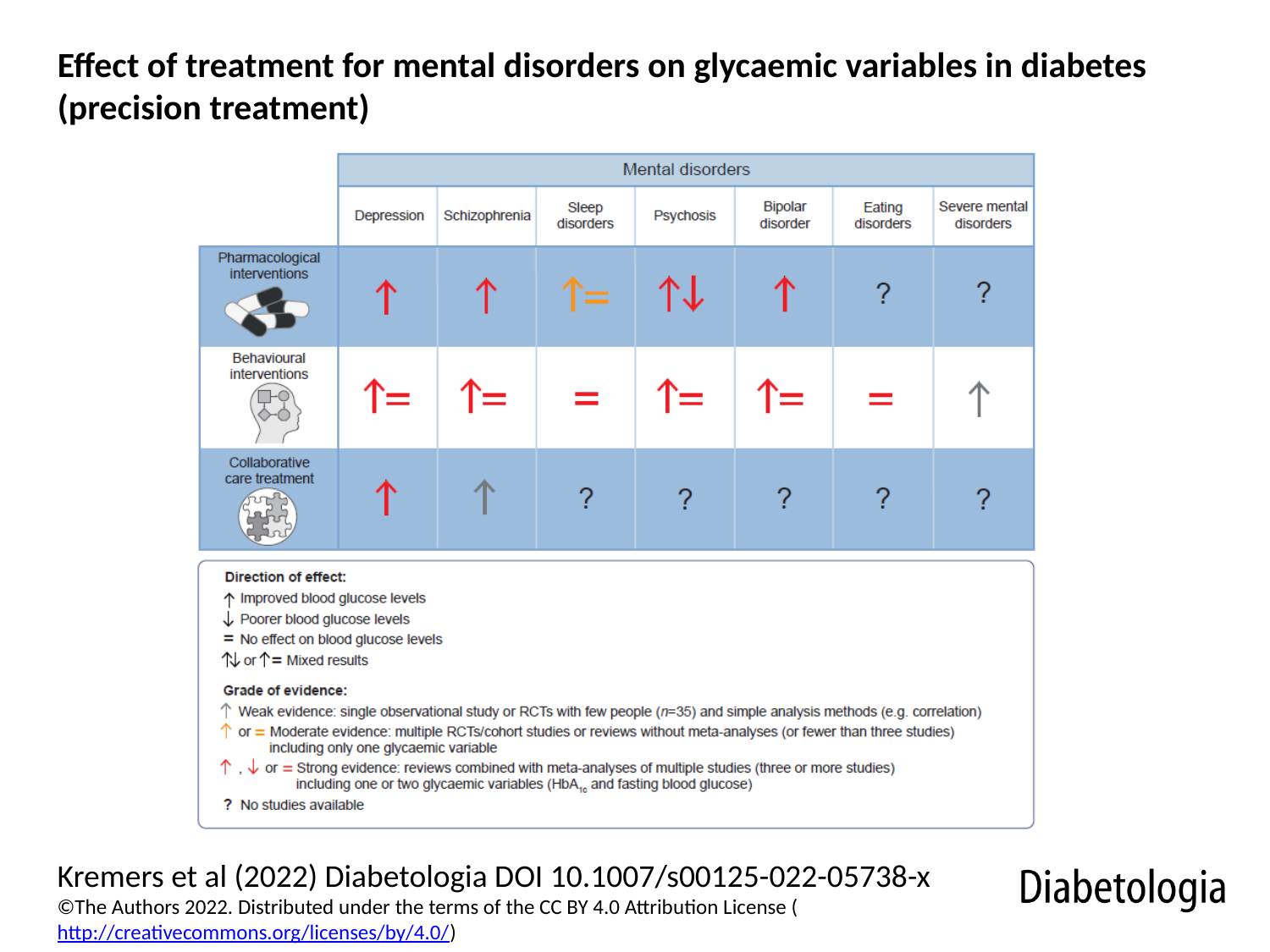

Effect of treatment for mental disorders on glycaemic variables in diabetes (precision treatment)
Kremers et al (2022) Diabetologia DOI 10.1007/s00125-022-05738-x
©The Authors 2022. Distributed under the terms of the CC BY 4.0 Attribution License (http://creativecommons.org/licenses/by/4.0/)
